# The Characterisation of Carbapenem-Resistant *Acinetobacter baumannii* and *Klebsiella pneumoniae* in a Teaching Hospital in Malaysia

**DOI:** 10.3390/antibiotics13111107

**Published:** 2024-11-20

**Authors:** Min Yi Lau, Sasheela Ponnampalavanar, Chun Wie Chong, Jacky Dwiyanto, Yee Qing Lee, Jia Jie Woon, Zhi Xian Kong, Azmiza Syawani Jasni, Michelle Chin Chin Lee, Unaizah Hanum Obaidellah, Cindy Shuan Ju Teh

**Affiliations:** 1 Department of Medical Microbiology, Faculty of Medicine, Universiti Malaya, Kuala Lumpur 50603, Malaysia; minyi@um.edu.my (M.Y.L.); jacky.dwiyanto@um.edu.my (J.D.); qing191093@gmail.com (Y.Q.L.); jiajiewoon93@hotmail.com (J.J.W.); kongzhixian@um.edu.my (Z.X.K.); 2 Infectious Disease Unit, Department of Medicine, Faculty of Medicine, Universiti Malaya, Kuala Lumpur 50603, Malaysia; 3 Department of Infectious Control, Universiti Malaya Medical Centre, Kuala Lumpur 50603, Malaysia; 4 School of Pharmacy, Monash University Malaysia, Subang Jaya 47500, Malaysia; chong.chunwie@monash.edu; 5 Faculty of Medicine and Health Sciences, Universiti Putra Malaysia, Serdang 43400, Malaysia; azmiza@upm.edu.my; 6 School of Psychology, Massey University, Albany, Auckland 0745, New Zealand; m.lee2@massey.ac.nz; 7 Department of Artificial Intelligence, Faculty of Computer Science and Information Technology, Universiti Malaya, Kuala Lumpur 50603, Malaysia; unaizah@um.edu.my

**Keywords:** carbapenem-resistant organisms, *Acinetobacter baumannii*, *Klebsiella pneumoniae*, epidemiology, antimicrobial-resistant

## Abstract

**Background/Objectives**: The emergence and dissemination of carbapenem-resistant organisms, particularly *Acinetobacter baumannii* and *Klebsiella pneumoniae*, pose a significant threat to healthcare systems worldwide. This retrospective study aims to characterise carbapenem-resistant *Acinetobacter baumannii* (CRAB) and carbapenem-resistant *Klebsiella pneumoniae* (CRKP) strains in a teaching hospital and to determine the risk factors associated with patients’ in-hospital mortality. **Methods**: A total of 90 CRAB and 63 CRKP were included in this study. Carbapenemase genes and MLST types of CRAB and CRKP were determined using specific primers. Risk factors associated with in-hospital mortality were analysed with collected data. **Results:** All the CRAB strains consisted of OXA carbapenemase genes, with 98% of the strains co-harbouring *bla*OXA-23-like and *bla*OXA-51-like carbapenemase genes. Conversely, *bla*NDM is the predominant carbapenemase gene in CRKP, followed by *bla*OXA-48-like carbapenemase genes. ST2 and ST20 are the dominant MLST types in CRAB and CRKP, respectively. In CRAB, multivariate analysis identified age, ethnicity, the presence of a mechanical ventilator, and patients who experienced previous exposure to clindamycin in the last 90 days as associated with an increased risk of in-hospital mortality. In contrast, older age, male, ICU admission, and the presence of an indwelling urinary catheter were significantly associated with an increased risk of mortality for patients with CRKP. **Conclusions**: Both CRAB and CRKP lead to high rates of mortality. The MLST profile showed that the genomic patterns of CRKP were highly diverse, whereas CRAB strains had low genetic diversity. To tackle these challenging pathogens, robust surveillance and an in-depth understanding of molecular epidemiology and genomics studies are needed to tailor infection control strategies and individualise treatment approaches.

## 1. Introduction

Carbapenem is commonly used as the ‘last line’ antibiotic to treat severe antimicrobial-resistant Gram-negative infections [1]. However, in recent decades, there has been a notable increase in the prevalence of Carbapenem-resistant organisms (CROs) worldwide, particularly carbapenem-resistant *Acinetobacter baumannii* (CRAB) and carbapenem-resistant *Klebsiella pneumoniae* (CRKP), highlighting a concerning trend in antimicrobial resistance [2]. CRAB and CRKP have been listed as critical pathogens in the World Health Organization (WHO) priority list of antibiotic-resistant bacteria [3]. Carbapenem-resistant *Enterobacterales* (CRE), *A. baumannii,* and ESBL *K. pneumoniae* are the top three healthcare-associated multidrug-resistant organisms (HA-MDRO) in Malaysia hospitals [4]. It has become a major concern for public health.

Several mechanisms drive carbapenem resistance among Gram-negative bacteria. These include the production of carbapenemases, the loss or alteration of outer membrane porin, the influence of efflux pump and hyperproduction, or the depression of Amp C β-lactamases [5]. In Gram-negative bacteria, the primary factor contributing to resistance is the presence of β-lactamases (carbapenemases) [6]. Class D carbapenemase (oxacillinase), including *bla*OXA-23-like, *bla*OXA-24-like, and *bla*OXA-58-like, are the most prevalent genes and formed the major cause of carbapenem resistance in *A. baumannii* [7]. In contrast, the key mechanisms contributing to carbapenem resistance in CRKP include all classes of carbapenemase, such as *Klebsiella pneumoniae* carbapenemase (KPC), New Delhi metallo-beta-lactamase (NDM), and OXA-type beta-lactamases [8]. These enzymatic activities disrupt the bactericidal action of carbapenems, leading to treatment failures and limited therapeutic options. Understanding the production of carbapenemase genes in CROs is crucial for developing effective strategies to combat the spread of this multidrug-resistant pathogen and ensure optimal patient care.

The number of CRKPs has doubled from 2016 to 2021, while an increase in CRAB has been observed in hospitals in Malaysia. According to the National Surveillance of Antimicrobial Resistance (NSAR) in Malaysia, the prevalence of *A. baumanii* resistant to meropenem has risen from 41.1% in 2018 to 63.3% in 2022 [9]. Besides that, the prevalence of CRKP resistant to meropenem also significantly increased from 0.3% in 2011 to 5.0% in 2022 [9]. Approximately 2500 CRE cases were reported in Malaysia hospitals in 2022, of which almost half were due to infection, and the mortality rate was 34% [10]. Despite the rising number of cases of CRAB and CRKP in Malaysia, comprehensive data related to its epidemiology remain scarce. To address this, we studied the epidemiology, resistance mechanisms, and risk factors associated with CRKP and CRAB in a tertiary teaching hospital in Kuala Lumpur. The result of this study will be crucial in devising effective infection control measures, optimising treatment strategies, and preventing further dissemination of these antibiotic-resistant strains in healthcare facilities and the community.

## 2. Results

### 2.1. Overview of CRAB Cases

A total of 90 CRAB strains were retrospectively collected in the years 2019 and 2020. The sample sources include wound swabs, sacral swabs, tracheal secretions, sputum, bronchoalveolar lavage, and others. This study comprised 90 patients, including individuals of Malay (26.7%), Chinese (28.9%), and Indian descent (36.7%), as well as other racial groups (7.8%), with 60 male (66.7%) and 30 female (33.3%) patients (Table 1). The mean age of the patients was 54.8, ranging from 1 month to 88 years old. A total of 42 patients (46.7%) passed away while receiving hospital care, and the remaining 48 patients recovered or were discharged from the hospital. A total of 36 (40.0%) patients were colonised with CRAB, and 54 (60.0%) were infected with CRAB.

Nearly 98% of the patients had been exposed to antibiotics in the past 90 days before the isolation of CRAB. The most common antibiotics were piperacillin-tazobactam (65.6%), meropenem (50.0%), augmentin/unasyn (42.2%), and vancomycin (42.2%). A total of 47 (87.0%) of the 54 infected patients were prescribed empiric antibiotics. Seven patients did not receive empiric treatment because they passed away on the day of diagnosis or the attending doctor opted not to treat the patient with systemic antibiotics. Meropenem (42.6%) and piperacillin-tazobactam (31.9%) were the frequently used empiric antibiotics treatment in this study. A total of 93.6% of treatment were inappropriate. Three patients received appropriate empiric treatment, whereby two patients received polymyxin B monotherapy and one patient received a combination of polymyxin B with meropenem. After obtaining culture results, 40 of 54 patients received definitive treatment, of which 36 (90.0%) were appropriate. This included polymyxin monotherapy (n = 5, 12.5%) and polymyxin combination therapy (n = 29, 72.5%). The combination therapy of polymyxin B and meropenem (n = 5, 12.5%) or polymyxin B and ampicillin-sulbactam (n = 18, 45.0%) was frequently used to treat CRAB in the hospital setting. Ten patients did not receive definitive treatment as they died or were discharged before microbiological susceptibility results were available. Three patients with superficial skin or soft tissue infection were not treated with systemic antibiotic.

A total of 90 CRAB patients with complete data were included in the R statistical analysis. Univariate analysis indicated that factors including age, ethnicity, the presence of a mechanical ventilator, cardiovascular disease, and previous exposure to clindamycin and meropenem were significantly associated with in-hospital mortality (*p* value < 0.1) and were included in the multivariate models (Table 2). The multivariate model showed that age, ethnic, mechanical ventilation, and patients who had previous exposure to clindamycin in last 90 days (AIC: 110.76) were associated with an increased risk of in-hospital mortality.

### 2.2. Overview of CRKP Cases

Sixty-three CRKP strains were collected from rectal swabs, blood, tracheal secretion, urine, sputum, pus, and aspirate. This study comprised 63 patients, including individuals of Malay (39.7%), Chinese (33.3%), and Indian descent (20.6%), as well as other racial groups (6.3%), with the majority of 39 male (61.9%) patients (Table 1). A total of 28.6% of the patients died during hospital admission. Forty-seven (74.6%) of the patients were colonised, whereas the remaining were infected (n = 16, 25.4%). Of the 63 patients, 48 (76.2%) were admitted to ICU during hospitalisation.

A total of 96.8% of the patients had exposure to antibiotics in the last 90 days prior to CRKP isolation. The most common antibiotics they were exposed to were piperacillin-tazobactam (52.4%), meropenem (46.0%), augmentin/unasyn (41.3%), and third-generation cephalosporin (34.9%). A total of 15 of the 16 infected patients received empiric antibiotics, of which 13 (86.7%) were inappropriate. Three patients received appropriate empiric treatment, with one polymyxin B and meropenem combination therapy and two meropenem therapies. After antibiotic susceptibility test results were available, 14 of the patients received definitive treatment, of which 10 (71.4%) were appropriate. One patient received polymyxin monotherapy, eight patients received polymyxin and meropenem combination therapy, and one patient received meropenem monotherapy appropriately. Four patients received inappropriate antibiotics such as meropenem, augmentin, and piperacillin-tazobactam. Two patients died after isolation, and therefore, no treatment was given.

Univariate analysis CRKP cases indicated that age, gender, ICU admission, the use of an invasive device (such as indwelling urinary catheter, mechanical ventilator, central venous catheter, and NG tubes), comorbidity (diabetes mellitus and malignancy), and previous exposure to vancomycin and macrolides were significantly associated with patient in-hospital mortality (*p*-value < 0.1). These predictors were then included in the multivariate analysis, which showed that gender; patients with invasive devices, including central venous catheters, mechanical ventilators, and NG tubes; patients with malignancy; and previous exposure to macrolides are associated with a high risk of in-hospital mortality. However, an infinity 95% CI was obtained in this model; hence, high-probability predictors, including the use of a mechanical ventilator, central venous catheter, NG tubes, and malignancy, were excluded from the multivariate analysis. The final multivariate model showed that a combination of older age, male patients, admission to ICU, and indwelling urinary catheters is significantly associated with increased risk of mortality (AIC 68.89).

### 2.3. Determination of Carbapenemase Genes Among CRAB and CRKP Strains

All CRAB strains harboured at least one carbapenemase gene, with nearly 98% co-harbouring dual carbapenemase genes *bla*OXA-23 and *bla*OXA-51. The *bla*OXA-58 was detected in 2 of the 90 CRAB strains. It is noteworthy that the carbapenem-hydrolysing Class D lactamase (CHDL) genes were the only carbapenemase genes found in *A. baumannii* isolated from UMMC.

Among 63 CRKP strains, 73% harbour carbapenemase genes, with *bla*NDM (71.7%) as the predominant carbapenemase gene, followed by *bla*OXA-48 (6.3%). No carbapenemase genes were detected in 17 CRKP strains (27%). *bla*KPC, *bla*VIM, and *bla*IMP were not detected in this study, but one CRKP strain that carried dual carbapenemase genes (*bla*NDM + *bla*OXA48) was detected.

### 2.4. Genetic Relatedness of CRAB and CRKP Strains

**Figure 1:**
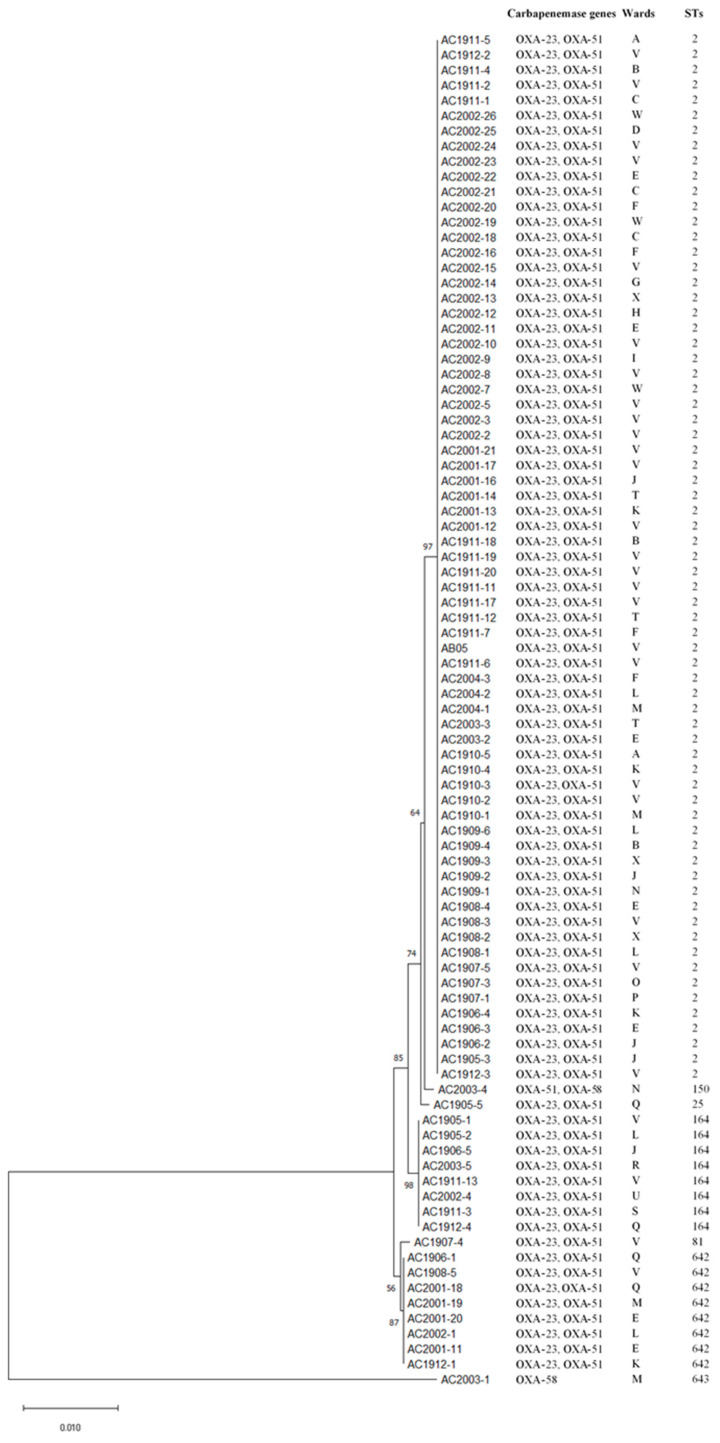
Phylogenetic tree of CRAB isolates.

ST2 (77.53%) was the dominant MLST type of CRAB in this study (Figure 1), followed by other STs, including ST164, ST642, ST25, ST81, ST643, and ST150.

**Figure 2:**
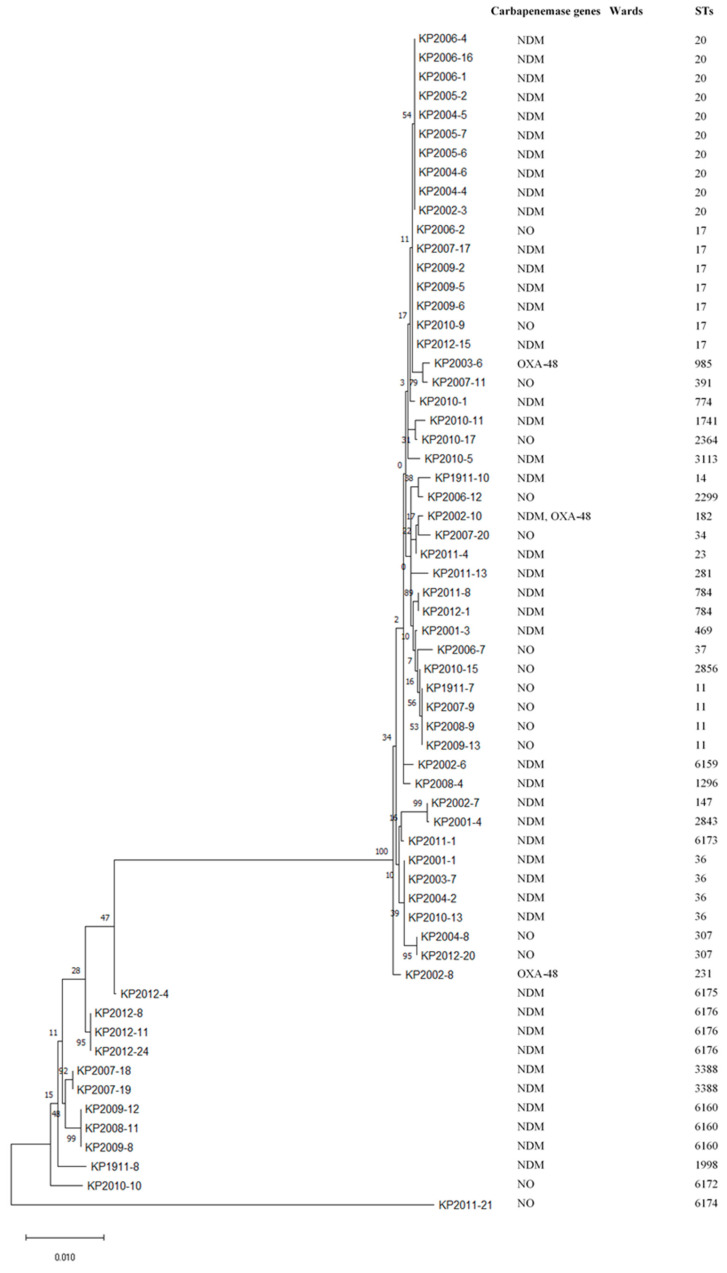
Phylogenetic tree of CRKP isolates. NO: No carbapenemase genes.

The MLST profile of CRKP is genetically diverse as compared to the CRAB profile (Figure 2). ST20 (16.13%) was the most prevalent MLST type in CRKP strains, followed by ST17 (11.29%). Other STs included three or fewer strains in each ST. CRKP strains showed 35 different sequence types.

BURST analysis was conducted to visualise the MLST data set into groups of related strains and clonal complexes. STs were grouped together only if they shared five or more matches of the seven loci with at least one ST in the group. In CRAB strains, only one group was identified, which is ST642 and ST81 as DLV, whereas other STs were singletons. Six groups were identified for CRKP strains. All in all, 19 singletons were detected from the CRKP strains.

**Figure 3:**
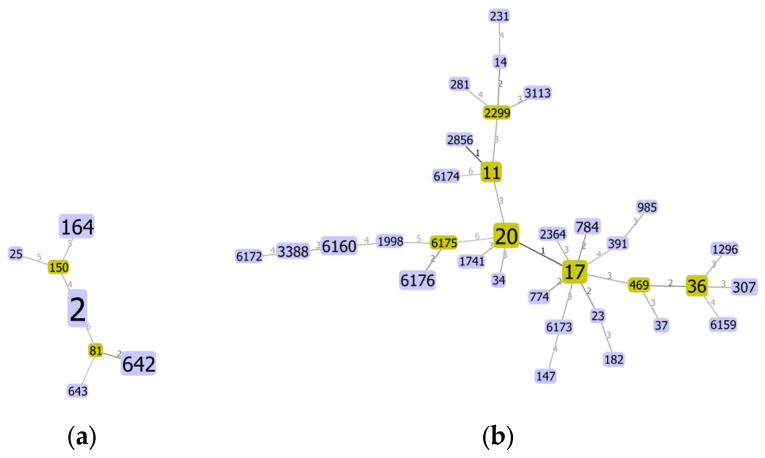
(**a**) Minimum spanning trees of CRAB strains. The ST prefix is not shown (i.e., 2 corresponds to ST2). (**b**) Minimum spanning tree of CRKP strains. The ST prefix is not shown (i.e., 20 corresponds to ST20).

The relationships between each ST are represented in MST (Figure 3). The numbers on the connecting line between each STs correspond to the numbers of allelic differences.

### 2.5. Antimicrobial Susceptibility Profiles and Modified Hodge Test

The complete antimicrobial susceptibility profiles for 84 CRAB and 62 CRKP strains are summarised in Table 3. All the CRAB strains were resistant to cefepime, imipenem, meropenem, and ampicillin-sulbactam, and more than 80% of the strains were resistant to amikacin, ceftazidime, ciprofloxacin, ceftriaxone, cefotaxime, and gentamicin. Ertapenem and trimethoprim-sulfamethoxazole showed activities against CRAB, with resistant rates of 78.57% and 64.29%, respectively.

All CRKP strains were resistant to amoxicillin-clavulanate, ampicillin, ampicillin-sulbactam, and piperacillin-tazobactam. However, these strains showed low resistance to amikacin (14.52%) and gentamicin (20.97%). CRKP strains showed more than 80% resistance to the remaining tested antibiotics, except for cefuroxime axetil (41.94%), cefepime (45.16%), cefoxitin (41.94%), imipenem (75.81%), and trimethoprim-sulfamethoxazole (74.19%).

Phenotypic carbapenemase detection using MHT was performed on the 63 CRKP strains. A total of 30 out of 63 strains were MHT-positive, 26 were negative, and 7 strains were undetected. Among the MHT-negative strains, 14 harboured carbapenemase genes.

## 3. Discussion

Carbapenem-resistant organisms (CROs) have been a significant problem for healthcare institutions worldwide for over two decades. The increase in the number of CRO cases in our hospital is of concern. Among the 90 patients with CRAB in this study, 58.8% were admitted into the ICU. All were hospital-acquired, of which 54 (60%) were infections, with an inpatient mortality rate of 57.4%, whereas 36 (40%) were colonised, with an in-hospital mortality rate of 30.6%. In this institution, 134 CRAB cases were reported in the general ICU in 2015 and 2016, with a mortality rate of more than 50% [11]. Additionally, an outbreak of CRAB was recorded in the Neonatal Intensive Care Unit (NICU) between December 2016 and February 2017, leading to the death of three neonates [12]. CRAB often tends to affect debilitated patients, especially those with underlying health conditions or compromised immune systems, those with invasive devices, and those managed in the ICU [13]. This may be one of the main contributing factors to the high mortality rate of patients with CRAB.

Enzymatic inactivation, porin alteration, and the actions of the efflux pump are recognised as important mechanisms of carbapenem resistance in *A. baumannii* [7,14]. Nevertheless, the production of enzymes, including metallo-β-lactamases and oxacillinases, continues to be the most frequent and widespread resistance mechanism in CRAB. Metallo-*β*-lactamase genes (MBLs), including *bla*NDM, *bla*IMP, and *bla*VIM, were absent in our CRAB strains. *bla*IMP was found in CRAB strains in this hospital nearly ten years ago; however, it has not been reported in recent studies [11,15]. MBLs are less commonly identified in *A. baumannii* compared to OXA-type carbapenemase [16]. All CRAB strains in this study harboured at least one OXA-type carbapenemase gene, and 98% of them harboured dual carbapenemases, with *bla*OXA-23-like and *bla*OXA-51-like as predominant carbapenemase genes. The prevalence rates in this study are in concordance with most of the studies in Malaysia and other Asian and Southeast Asian countries [16,17,18,19]. OXA-type carbapenemases, which are class D β-lactamases, have been reported as the most widespread carbapenemase in *A. baumannii,* especially *bla*OXA-23-like [7,14]. *bla*OXA-23-like is mainly plasmid-encoded, which allows for horizontal transfer and facilitates the spread of this gene to different bacterial populations [20]. *bla*OXA-23-like was the first OXA-type carbapenemase identified from CRAB and remains the most prevalent worldwide today [16]. This gene has been implicated in numerous outbreaks of CRAB in healthcare settings, particularly in ICUs [21,22]. *bla*OXA-51-like genes are found to be naturally occurring in *A. baumannii* and are chromosomally located, which may explain their high prevalence [7]. Both *bla*OXA-23-like and *bla*OXA-51-like are the most prevalent carbapenemases in CRAB worldwide. The phylogenetic tree in this study (Figure 1) suggests that these genes are persistent within the healthcare setting and may serve as an ongoing source of transmission in HAI. Studies have shown that these organisms can survive on inanimate surfaces for months if environment cleaning is poor [23]. Thus, continuous surveillance and improvement in infection control measures are pivotal in healthcare facilities to manage and prevent outbreaks [12].

In contrast, the prevalence of *bla*OXA-58 is considered low as compared to *bla*OXA-23-like and *bla*OXA-51-like in CRAB in the Asian Pacific [24]. *bla*OXA-58 is mostly reported in the Western hemisphere, especially France, Spain, Italy, and the US, but rarely reported in Malaysia and other Asian countries [19,25]. Based on a previous study, *bla*OXA-58 was absent in our hospital settings in 2015 and 2016 [11]. In this study, the emergence of *bla*OXA-58 in our hospital is concerning and requires immediate attention, as this gene is plasmid-mediated [26] and has been associated with various HAIs and implicated in outbreaks [27]. Further, it represents a new challenge in the treatment and management of carbapenem-resistant infections. It is worth mentioning that the sequence type of CRAB strains harbouring *bla*OXA-58 is ST150 and ST643 and is different from the majority gene (*bla*OXA-23-like and *bla*OXA-51-like), which is mainly ST2. ST2 (77.5%) was our hospital’s dominant MLST type of CRAB. ST2 was the predominant sequence type of CRAB reported in Malaysia [19,28,29] and was also reported as the most prevalent sequence type of CRAB in Southeast Asia [30] and the United States [31]. A comparison between MLST profiles of all CRAB strains in this study showed low genetic diversity, with only seven sequence types detected among 90 CRAB strains. The presence of a dominant genotype among 77.5% of strains suggested the clonal spread of this pathogen in the hospital, emphasising the need to improve infection control measures.

Based on the 2022 National Antimicrobial Utilization Report, the use of broad-spectrum antibiotics is higher in teaching hospitals [9]. Once *A. baumannii* exhibits carbapenem resistance, it generally has acquired resistance to most other antibiotics, limiting its treatment options [32]. Furthermore, current treatment options and regimens are based on limited and conflicting data [33]. In resource-limited settings such as ours, where newer therapeutic options are not available, high-dose ampicillin-sulbactam in combination with polymyxin B is usually used [34,35]. It has been reported that a combination of polymyxin and ampicillin-sulbactam demonstrated high synergistic activity in vitro, especially in colistin-resistant strains [36]. However, the use of polymyxin is associated with an increased risk of nephrotoxicity [37]. The resistance rate of polymyxin B is also increasing, reported at 5.6% in 2022 [9]. The mortality rate associated with CRAB infections remains high (30.6%) even with appropriate polymyxin B treatment [38]. In this study, 3 out of 5 (60%) patients who received polymyxin B monotherapy and 15 out of 29 (51.7%) who received combination therapy died during hospitalisation.

Risk factors of mortality in patients with CRAB in this study include older age, Malay descent, the presence of a mechanical ventilator, and previous exposure to clindamycin. A total of 46.7% (n = 42) of patients died during their hospital stay. Infections with CRAB in healthcare settings are often seen in patients undergoing invasive procedures [35]. The contamination of mechanical ventilators has been reported as a source of outbreaks leading to high mortality [39,40]. Biofilm-forming *A. baumannii* contributes to its persistence in invasive devices and hospital environments [41]. The hydrophobic ability enables this organism to survive on abiotic surfaces and is hard to eliminate, even after four rounds of routine cleaning and disinfection [42]. Low immunity change in microbiota [43] may predispose older and ventilated patients to CRAB infection and an increased mortality rate. A specific link between ethnicity and CRAB infections was not established. Additionally, the small sample size in this study limits the ability to draw definitive conclusions about this factor. A further study on host genetic contributions to susceptibility to CRO infection is needed.

Interestingly, patients with prior exposure to clindamycin showed a lower risk of mortality in this study. Clindamycin, which is a lincosamide compound, is often used for anaerobic bacteria infection and has limited use as a single antimicrobial agent in *A. baumannnii* infections. A study of the activity of the efflux pump inhibitor phenylalanine-arginine β-naphthylamide (PaβN) against the AdeFGH pump of *A. baumannii* showed that PaβN had a greater inhibitory effect on the efflux of clindamycin compared to trimethoprim and chloramphenicol mediated by the AdeFGH efflux pump in *A. baumannii* [44]. Clindamycin can prevent the derepression of β-lactamases in certain bacterial strains without affecting the synthesis of other proteins or the bacterial replication process [45]. This suggests the potential of clindamycin as a unique therapeutic agent that not only directly targets bacterial growth but also disrupts the mechanisms of antibiotic resistance, particularly by inhibiting the induction of β-lactamase production. However, only a few patients had previous exposure to clindamycin in this study, and further analysis is required when more cases are included in the future.

In this study, 63 CRKP were collected, of which 47 were carbapenemase-producing CRKP (CP-CRKP), and 16 were non-carbapenemase-producing CRKP (NC-CRKP), with a mortality rate of 28.6%. Based on the trend in UMMC, the number of CRKP strains gradually increased from 17 strains in 2013 [46] to 115 strains in 2015 [47]. Importantly, there was an overall increase in CRKP, encompassing both CP-CRKP and NC-CRKP strains [48]. The only carbapenemase genes detected in this study are *bla*NDM (68.3%) and *bla*OXA-48-like (6.3%). Our previous studies suggest that the prevalence and persistence of *bla*NDM and *bla*OXA-48-like carbapenemase genes in our hospital setting have remained consistent over time [49]. However, the trend of CRKP has changed. Based on previous reports, the predominant carbapenemase gene in our hospital setting is the *bla*OXA-48-like gene [46,47,49]. Data suggested that *bla*NDM has replaced *bla*OXA-48-like as the predominant carbapenemase gene in CRKP strains in UMMC. This is not surprising as *bla*NDM predominates in Asia [50] and is leading cause of CRE in Malaysia [10]. Although NDM producers originated from the Indian subcontinent, their rapid dissemination worldwide, including Western Europe, North America, and the Far East, has been reported [8]. The increased prevalence of *bla*NDM among CRKP strains implied that the high transmissibility and plasticity of *bla*NDM-positive plasmids might have contributed substantially to its spread among existing bacterial populations in UMMC. IncL and IncFIIK plasmids carried by *bla*OXA-48 and *bla*NDM contributed to the conjugation and high plasmid stability of these genes [51]. Recently, the high prevalence of *bla*OXA-48 and *bla*NDM has been reported in Iran and Southern California [52,53,54]. This was similar to the carbapenemase trends observed in Malaysia. In this study, a *K. pneumoniae* strain carrying dual carbapenemase genes was identified, as previously reported [49]. Strains with dual carbapenemase genes are associated with higher levels of carbapenem resistance, which can lead to treatment complications and increased morbidity and mortality [55]. Other major carbapenemase genes, such as *bla*KPC, *bla*IMP, and *bla*VIM, have been reported in Malaysia but were absent in this study [46,47,56]. *bla*KPC was mainly reported in North America and slowly disseminated to other European countries but still remains less prevalent in Asian continents, whereas *bla*VIM was frequently isolated in Mediterranean countries. *bla*IMP was primarily described in Japan; however, the dissemination of IMP-producing *K. pneumoniae* in the rest of the world appears to be limited [8].

MLST analysis revealed that 34 sequence types were detected among 63 CRKP strains, indicating greater diversity in CRKP compared to CRAB strains. ST20 (16.13%) was the most prevalent ST in CRKP strains in this study, which is in contrast to 2017, where the predominant ST in our hospital was ST101 [46]. This demonstrates that the genomic patterns of CRKP can be highly diverse and dynamic, even within the same hospital setting. MLST analysis revealed genetic diversity among the CRKP strains, highlighting their complex and heterogeneous nature. This diversity indicates that multiple different lineages of *K. pneumoniae* are circulating and contributing to carbapenem resistance within the hospital and likely arising from multiple independent sources circulating within the hospital rather than from a single clonal outbreak.

Based on the previous report, the in-hospital mortality rate of CRKP in 2013, 2014, 2015, and 2016 to 2017 was 35.3%, 27.3%, 42.6%, and 43.4%, respectively [46,47,49]. In this study, the rate was higher at 73%. However, this may be an overestimation and may not reflect the annual mortality rate. Nevertheless, one of the main reasons for the high mortality of CRKP in this region is the limited effective treatment options. The first-line therapy that is mostly used in resource-limited settings is polymyxin combination therapy, which has been shown to be significantly inferior to newer β-lactam/β-lactamase inhibitor (BLBI) combinations, like ceftazidime-avibactam (CAZ/AVI) and meropenem-vaborbactam, in randomised controlled trials [57]. Unfortunately, many of these trials have focused on KPC-producing CRE, and their efficacy against metallo-β-lactamase (MBL)-producing strains like NDM is limited, necessitating the exploration of other combination therapies. In a prospective study by Falcone et al., the mortality rate was significantly lower when using a combination of ceftazidime-avibactam (CAZ/AVI) with aztreonam (9.2%) compared to polymyxin-based combination therapy (44%) for treating NDM-producing *Enterobacterales* [58]. However, the ability of these antibiotics in resource-limited settings is often limited due to high costs, a lack of approval, or restricted access.

Older age, male, ICU admission, and indwelling urinary catheters are significantly associated with an increased risk of mortality. Patients who were admitted to the ICU have more severe illnesses, numerous comorbidities, and invasive devices; are often immunocompromised; and have a greater susceptibility to infections. Therefore, they are exposed to more antibiotics [59]. Frequent invasive procedures and indwelling tubes can damage the mucosal barrier, leading to a further increased risk of CRKP exogenous and endogenous infection [60]. CRKP can form biofilms on the surfaces of indwelling devices, making the infection more difficult to treat and more persistent, leading to a higher risk of in-hospital mortality [61].

The limitation of the current study is the limited number of selected CROs from a teaching hospital due to limited resources. Hence, not all CROs are included; the small sample size may include potential bias and lead to deviation in statistical analysis. This study could only represent the current assessment of CRAB and CRKP in our hospital at the present time. In this study, several confounders, such as polymicrobial infections and other underlying diseases, might affect the risk factor of in-hospital mortality analysis.

## 4. Materials and Methods

### 4.1. Ethics Statement

The medical ethics approval was obtained from the Universiti Malaya Medical Centre Medical Ethics Committee before extracting clinical data from the hospital database (MREC No: 2020117-8191).

### 4.2. Bacterial Strain Collection and Hospital Setting

This retrospective study was conducted at the Universiti Malaya Medical Centre (UMMC), a 1600-bed tertiary public teaching hospital in Kuala Lumpur, Malaysia, from May 2019 to December 2020. In 2019, there were 155 reported cases of CRAB and 66 of CRE. In 2020, the reported cases increased to 161 for CRAB and 140 for CRE. Approximately 30% of the strains from both years, including 90 CRAB and 63 CRKP, were randomly selected. The selected strains were revived from the collection in the microbiology laboratory and were included for analysis in this study. CRAB and CRKP that exhibited resistance or intermediate resistance to at least one carbapenem (imipenem, meropenem or ertapenem), as determined by Clinical and Laboratory Standards Institute (CLSI) 2017 guidelines, were included in this study [62]. The strains were collected from various samples, including swabs, sputum, tracheal secretions, bronchoalveolar lavage, urine, blood, and tissue. All bacterial strains were revived from glycerol stock culture and checked for purity before the commencement of benchwork. Crude DNA was extracted by using the direct boiling method [49].

### 4.3. Detection of Carbapenemase Genes

The presence of carbapenemase genes in CRAB, which included *bla*OXA [63], *bla*IMP [64], *bla*VIM [65], and *bla*NDM [66], and carbapenemase genes in CRKP such as *bla*KPC [67], *bla*OXA-48-like [68], *bla*IMP, *bla*VIM, and *bla*NDM [69], were detected by using polymerase chain reaction (PCR) with specific primers and cycling parameters, as previously described. The amplicon was then analysed using 1.0% agarose gel.

### 4.4. Clonal Relatedness of Strains

Multi-locus sequence typing (MLST) was performed on CRAB and CRKP strains by using Pasteur Institute scheme (http://pubmlst.org/abaumannii/, accessed on 14 February 2023 and http://www.pasteur.fr/mlst/, accessed on 15 February 2023) [70,71,72]. Seven housekeeping genes for each species (CRAB: *cpn60*, *fusA*, *gltA*, *pyrG*, *recA*, *rplB*, *rpoB*; CRKP: *gapA*, *infB*, *mdh*, *pgi*, *phoE*, *rpoB*, *tonB*) were amplified and sequenced to determine the sequence types and clonal relatedness among strains. Phylogenetic trees were constructed with the maximum likelihood method by using MEGA software version 10.1.7 [73]. The differences in allelic profiles were determined by using the BURST plugin with BIGSdb database software in PubMLST (https://pubmlst.org/software/bigsdb, accessed on 12 January 2024) [70]. Allelic profiles that differ from one another at one of the seven MLST loci are called single-locus variants (SLVs), two of the seven loci are called double-locus variants (DLVs), and three of the seven loci are called triple-locus variants (TLVs) [74]. STs that cannot be assigned to any groups are defined as singletons. A minimum spanning tree (MST) was created with the goeBURST algorithm in PHYLOViS 2.0 software to identify the clonal complexes (CC) [74,75]. Untypeable strains were removed from the analysis.

### 4.5. Antimicrobial Susceptibility Testing and Modified Hodge Test

Bacterial identification and antimicrobial susceptibility testing (AST) were performed by an automated system (Vitek^®^ 2; BioMérieux, Marcy L’Etoile, France). A total of 13 antibiotics were tested for CRAB strains, including amikacin (AN), ceftazidime (CAZ), ciprofloxacin (CIP), ceftriaxone (CRO), cefotaxime (CTX), ertapenem (ETP), cefepime (FEP), gentamicin (GM), imipenem (IPM), meropenem (MEM), ampicillin-sulbactam (SAM), trimethoprim-sulfamethoxazole (SXT), and piperacillin-tazobactam (TZP). In contrast, five additional antibiotics, which consist of amoxicillin-clavulanate (AMC), ampicillin (AMP), cefuroxime (CXM), cefuroxime axetil (CXMA), and cefoxitin (FOX), were tested for CRKP strains. AST was determined by using the AST-N314 card (BioMérieux, Marcy L’Etoile, France) under the Vitek 2 system. Susceptibility breakpoints were interpreted based on the recommendations of the CLSI 2017 (http://www.clsi.org/). Colistin susceptibility test was not included because broth microdilution was the only recommended testing standard for colistin by CLSI and the European Committee on Antimicrobial Susceptibility Testing (EUCAST).

The detection of carbapenemase production in *K. pneumoniae* was carried out using the Modified Hodge Test (MHT), with *K. pneumoniae* ATCC BAA-1705 and *K. pneumoniae* ATCC BAA-1706 being utilised as MHT-positive and -negative controls, respectively, according to CLSI guideline [62].

### 4.6. Clinical Data Collection and Statistical Analysis

All patients’ data were retrieved from the electrical medical record (EMR), including patient demographics, length of hospital stay, isolation date, sample sources, previous hospitalisation, intensive care unit (ICU) admission, presence of invasive device, comorbidity, antimicrobial exposure within 90 days of CROs isolation, empirical treatment, definitive treatment, and treatment outcome. An empiric antibiotic is defined as the initial antibiotic treatment given within 48 h of obtaining culture, prior to the availability of microbiological results. The empiric antibiotic was deemed appropriate if its spectrum included coverage for CRAB or CRKP [76,77]. Definitive treatment is defined as antibiotics administered to the patient based on the microbiological susceptibility results once they are available [77]. Patients were classified as having an infection when clinical and biochemical evidence of infection was present, while patients who exhibited no signs or symptoms were categorised as colonisation [78]. The location of acquisition of CROs is categorised as follows: (1) Hospital-acquired infection (HAI), which is defined as the isolation of a microorganism from patients more than 48 h after hospitalisation who did not show any signs or symptoms during admission [79]. (2) Community-acquired infection (CAI), which is defined as the isolation of microorganisms from patients less than 48 h after admission who showed symptoms and did not come into contact with healthcare facilities in the past 3 months [79]. (3) Healthcare-associated infection (HCAI), which refers to the isolation of microorganisms from patients less than 48 h after admission who had contact with healthcare in the previous three months [79,80]. (4) Colonisation (HAC) is defined as the isolation of microorganisms from any non-sterile body site with the absence of clinical findings of infection [81].

Risk factors associated with in-hospital mortality were first compared using IBM SPSS™ Statistic software (New York, NY, USA), version 28. A chi-square test or Fisher’s exact test was performed for categorical variables. In contrast, continuous variables were analysed using either the Mann–Whitney U test or Student’s t-test based on data normality. All statistical analysis was further conducted in R version 4.2.2. Completed clinical data for 90 CRAB and 63 CRKP strains were included in the analysis. A collinearity test was carried out to test the reliability of regression coefficients. Logistic regression analysis using the ‘glmer()’ function was conducted in R to determine the risk factors associated with in-hospital mortality with CROs. Empiric and definitive treatments were analysed with infected patients only. Variables with a *p* value less than 0.1 were selected for inclusion in multivariate logistic regression analysis, which run in the build model in the R package. ‘Dredge’ command was implemented in the R MuMIn package to identify the parsimonious model.

## 5. Conclusions

In conclusion, OXA-type carbapenemase genes were the only carbapenemases detected in CRAB strains in this study. *bla*OXA-58, which is rare in local findings, was detected in this study. In contrast, the trend of CRKP strains in this hospital changed from *bla*OXA-48 into the *bla*NDM carbapenemase gene. The dominant sequence types of CRAB and CRKP are ST2 and ST20, respectively. The squence type of CRKP is more diverse than CRAB in this study. Early detection, effective preventive measures, and the development of novel agents with reliable clinical efficacy are crucial in addressing the spread of CROs. The findings in this study may assist support the prevalence and epidemiology data of CROs in the hospital in Malaysia. The identification of carbapenem-resistant genes assisted in selecting appropriate antibiotic treatment and guiding future interventions and infection control of CROs in Malaysia.

## Figures and Tables

**Table 1:** Risk factors for in-hospital mortality for patients with CRAB and CRKP isolation.

Risk Factors	In-Hospital Mortality CRAB		Chi Square (*p* Value)	In-Hospital Mortality CRKP		Chi Square (*p* Value)
	No (n = 48)	Yes (n = 42)		No (n = 45)	Yes (n = 18)	
**Gender**			1			0.027 *
Female	16 (53.3%)	14 (46.7%)		21 (87.5%)	3 (22.5%)	
Male	32 (53.3%)	28 (46.7%)		24 (61.5%)	15 (38.5%)	
**Ethnic**			0.106			0.645
Malay	9 (37.5%)	15 (62.5%)		16 (64.0%)	9 (36.0%)	
Chinese	16 (61.5%)	10 (39.5%)		17 (81.0%)	4 (19.0%)	
India	17 (51.5%)	16 (48.5%)		9 (69.2%)	4 (30.8%)	
Others	6 (85.7%)	1 (14.3%)		3 (75.0%)	1 (25.0%)	
**ICU admission**	26 (49.1%)	27 (50.9%)	0.330	31 (64.6%)	17 (35.4%)	0.047 *^a^
**Infection acquire model**			0.021 *			0.292
CAI	0 (0%)	0 (0%)		0 (0%)	0 (0%)	
HAI	23 (44.2%)	29 (55.8%)		8 (57.1%)	6 (42.9%)	
HAC	25 (69.4%)	11 (30.6%)		36 (76.6%)	11 (23.4%)	
HCAI	0 (0%)	2 (100%)		1 (50%)	1 (50%)	
**Invasive device**						
Indwelling urinary catheter	41 (50.6%)	40 (49.4%)	0.166 ^a^	32 (65.3%)	17 (34.7%)	0.051 ^a^
Mechanical ventilator	33 (47.1%)	37 (52.9%)	0.028 *	32 (64.0%)	18 (36.0%)	0.013 *^a^
Tracheostomy	13 (54.2%)	11 (45.8%)	0.924	10 (71.4%)	4 (28.6%)	1.000 ^a^
Central veneous catheter	35 (49.3%)	36 (50.7%)	0.138	26 (59.1%)	18 (40.9%)	<0.001 *^a^
Art line	26 (49.1%)	27 (50.9%)	0.330	28 (65.1%)	15 (34.9%)	0.104
Peripheral veneous line	40 (52.6%)	36 (47.4%)	0.756	40 (71.4%)	16 (28.6%)	1.000
NG tube	36 (50.0%)	36 (50.0%)	0.205	27 (60.0%)	18 (40.0%)	0.001 *^a^
Stoma	4 (57.1%)	3 (42.9%)	1.000 ^a^	4 (80.0%)	1 (20.0%)	1.000 ^a^
**Specimen sources**			0.048 *			0.197
Respiratory	30 (46.2%)	35 (53.8%)		7 (77.8%)	2 (22.2%)	
Urinary	2 (100%)	0 (0%)		4 (100%)	0 (0%)	
Blood	1 (25.0%)	3 (75.0%)		5 (83.3%)	1 (16.7%)	
Skin and soft tissue	14 (77.8%)	4 (22.2%)		5 (100%)	0 (0%)	
Rectal swabs	0 (0%)	0 (0%)		23 (63.9%)	13 (36.1%)	
Others	1 (100%)	0 (0%)		1 (33.3%)	2 (66.7%)	
**Previous hospitalisation**						
>1 year	2 (100%)	0 (0%)	0.497 ^a^	2 (66.7%)	1 (33.3%)	1.000 ^a^
6–12 months	5 (55.6%)	4 (44.4%)	1.000 ^a^	4 (100%)	0 (0%)	0.317 ^a^
3–6 months	2 (33.3%)	4 (66.7%)	0.412 ^a^	4 (100%)	0 (0%)	0.317 ^a^
<3 months	26 (61.9%)	16 (38.1%)	0.127	27 (71.1%)	11 (28.9%)	0.935
none	13 (48.1%)	14 (51.9%)	0.519	8 (61.5%)	5 (38.5%)	0.492 ^a^
**Previous contact to health care facilities**	29 (53.7%)	25 (46.3%)	0.931	34 (72.3%)	13 (27.7%)	0.760 ^a^
**Comorbidity**						
CKD	19 (45.2%)	23 (54.8%)	0.150	23 (67.6%)	11 (32.4%)	0.472
DM	22 (47.8%)	24 (52.2%)	0.284	19 (61.3%)	12 (38.7%)	0.080
CVD	8 (36.4%)	14 (63.6%)	0.066	9 (56.3%)	7 (43.8%)	0.198 ^a^
Malignancy	14 (63.6%)	8 (36.4%)	0.265	7 (100%)	0 (0%)	0.177 ^a^
HPT	20 (46.5%)	23 (53.5%)	0.215	24 (64.9%)	13 (35.1%)	0.169
**Previous antibiotic exposure in last 90 days**	48 (54.5%)	40 (45.5%)	0.215 ^a^	43 (70.5%)	18 (29.5%)	1.000 ^a^
Conventional Penicillin	8 (72.7%)	3 (27.3%)	0.169	3 (75.0%)	1 (25.0%)	1.000 ^a^
Cloaxacillin	5 (55.6%)	4 (44.4%)	1.000 ^a^	9 (81.8%)	2 (18.2%)	0.489 ^a^
Augmentin/Unasyn	17 (44.7%)	21 (55.3%)	0.162	20 (76.9%)	6 (23.1%)	0.418
Piperacillin-tazobactam	31 (52.5%)	28 (47.5%)	0.836	22 (66.7%)	11 (33.3%)	0.380
Aminoglycoside	6 (60.0%)	4 (40%)	0.745 ^a^	3 (75.0%)	1 (25.0%)	1.000 ^a^
Vancomycin	23 (60.5%)	15 (39.5%)	0.242	9 (52.9%)	8 (47.1%)	0.063 ^a^
1st-generation cephalosporin	0 (0%)	1 (100%)	0.467 ^a^	4 (100%)	0 (0%)	0.317 ^a^
2nd-generation cephalosporin	9 (56.3%)	7 (43.7%)	0.796	7 (87.5%)	1 (12.5%)	0.421 ^a^
3rd-generation cephalosporin	17 (63.0%)	10 (37.0%)	0.231	16 (72.7%)	6 (27.3%)	0.867
4th-generation cephalosporin	5 (50.0%)	5 (50.0%)	1.000 ^a^	6 (60.0%)	4 (40.0%)	0.452 ^a^
5th-generation cephalosporin	1 (100%)	0 (0%)	1.000 ^a^	-	-	-
Clindamycin	8 (88.9%)	1 (11.1%)	0.033 ^a^	2 (100%)	0 (0%)	1.000 ^a^
Metronidazole	10 (55.6%)	8 (44.4%)	0.833	5 (71.4%)	2 (28.6%)	1.000 ^a^
Polymyxin B	1 (20.0%)	4 (80.0%)	0.181 ^a^	4 (66.7%)	2 (33.3%)	1.000 ^a^
Meropenem	20 (44.4%)	25 (55.6%)	0.091	21 (72.4%)	8 (27.6%)	0.873
Macrolides	6 (46.2%)	7 (53.8%)	0.575	7 (100%)	0 (0%)	0.177 ^a^
Fluoroquinolones	2 (40.0%)	3 (60.0%)	0.661 ^a^	2 (100%)	0 (0%)	1.000 ^a^
Bactrim	2 (40.0%)	3 (60.0%)	0.661 ^a^	2 (50.0%)	2 (50.0%)	0.571 ^a^
**Monoinfection**	6 (40.0%)	9 (60.0%)	0.257	10 (76.9%)	3 (23.1%)	0.741 ^a^
**Carbapenemase genes**						
OXA-23	47 (53.4%)	41 (46.6%)	1.000 ^a^	-	-	-
OXA-51	47 (52.8%)	42 (47.2%)	1.000 ^a^	-	-	-
OXA-58	1 (50.0%)	1 (50.0)	1.000 ^a^	-	-	-
OXA-48				3 (75.0%)	1 (25.0%)	1.000 ^a^
NDM				31 (72.1%)	12 (27.9%)	0.864
No carbapenemase				12 (75.0%)	4 (25.0%)	1.000 ^a^
**Empiric antibiotics** **(Patients received treatmemt: CRAB = 47; CRKP = 15)**	21 (44.7%)	26 (55.3%)	0.685 ^a^	9 (60.0%)	6 (40.0%)	0.438 ^a^
**Appropriate treatment (CRAB = 3; CRKP = 3)**	1 (33.3%)	2 (66.7%)	1.000 ^a^	1 (33.3%)	2 (66.7%)	0.525 ^a^
Polymyxin B monotherapy	1 (50.0%)	1 (50.0%)	1.000 ^a^	-	-	-
Polymyxin B and Meropenem therapy	0 (0.0%)	1 (100.0%)	1.000 ^a^	1 (100.0%)	0 (0.0%)	1.000 ^a^
Meropenem	-	-	-	0 (0.0%)	2 (100.0%)	0.143 ^a^
**Inappropriate treatment**	20 (45.5%)	24 (54.5%)	1.000 ^a^	8 (66.7%)	4 (33.3%)	0.525 ^a^
**Definitive Treatment** **(Patients received treatment: CRAB = 40; CRKP = 14)**	19 (47.5%)	21 (52.5%)	0.218	9 (64.3%)	5 (35.7%)	0.175 ^a^
**Appropriate treatment (CRAB = 36; CRKP = 10)**	18 (50.0%)	18 (50.0%)	0.607 ^a^	6 (60.0%)	4 (40.0%)	1.000 ^a^
Polymyxin B monotherapy	2 (40.0%)	3 (60.0%)	1.000 ^a^	1 (100.0%)	0 (0.0%)	1.000 ^a^
Polymyxin B and Unasyn therapy	10 (43.5%)	8 (44.4%)	0.356	-	-	-
Polymyxin B and Meropenem therapy	2 (22.2%)	7 (77.8%)	0.133 ^a^	5 (62.5%)	3 (37.5%)	1.000 ^a^
Polymyxin B and other therapy	2 (100.0%)	0 (0.0%)	0.219 ^a^	-	-	-
Ciprofloxacin	1 (100.0%)	0 (0.0%)	0.475 ^a^	-	-	-
Ceftazidime	1 (100.0%)	0 (0.0%)	0.475 ^a^	-	-	-
Meropenem	-	-	-	0 (0.0%)	1 (100.0%)	1.000 ^a^
**Inappropriate treatment**	1 (25.0%)	3 (75.0%)	0.607 ^a^	3 (75.0%)	1 (25.0%)	1.000 ^a^

* *p* < 0.05. ^a^ *p* value obtained using Fisher’s exact test. CKD: chronic kidney disease; DM: diabetes mellitus; CVD: cardiovascular disease; HPT: hypertension.

**Table 2:** Logistic regression analysis for mortality associated with CRAB and CRKP infection.

Risk Factors	CRAB (Total Infected Patients = 54)		Odd Ratio (95% CI)	CRKP (Total Infected Patients = 16)		Odd Ratio (95% CI)
	Univariate Analysis	Multivariate Analysis		Univariate Analysis	Multivariate Analysis	
**Gender**	1.000			0.027	0.073 [0.015]	3.86 (0.88–16.93) [8.75 (1.53–50.11)]
**Age**	0.020	0.026	0.10 (0.01–0.76)	0.036	0.088	1.04 (0.99–1.08)
**Ethnic**	0.083	0.062	0.60 (0.35–1.03)	0.582		
**ICU admission**	0.336			0.032	0.160	5.10 (0.52–49.59)
**Infection acquire model**	0.155			0.356		
**Invasive device** Indwelling urinary catheter	0.124			0.045	0.121	5.48 (0.57–52.77)
Mechanical ventilator	0.028	0.007	5.16 (1.56–17.09)	0.001	[0.997]	[5.92 (0–Inf)]
Tracheostomy	0.925			1.000		
Central veneous catheter	0.141			0.001	[0.997]	[4.80 (0–Inf)]
Art line	0.335			0.107		
Peripheral veneous line	0.759			1.000		
NG tube	0.209			0.001	[0.996]	6.71 [(0–Inf)]
Stoma	0.835			0.665		
**Previous hospitalisation**						
<3 months	0.130			0.936		
none	0.524			0.384		
**Previous contact to health care facilities**	0.932			0.788		
**Comorbidity**						
CKD	0.153			0.480		
DM	0.290			0.082		
CVD	0.068			0.124		
Malignancy	0.270			0.078	[0.997]	[5.92 (0–Inf)]
HPT	0.219			0.174		
Dyslipidemia	-			0.185		
**Previous antibiotic exposure in last 90 days**						
Conventional Penicillin	0.173			-		
Cloaxacillin	0.890			0.409		
Augmentin/Unasyn	0.170			0.427		
Piperacillin-tazobactam	0.838			0.388		
Aminoglycoside	0.658			-		
Vancomycin	0.247			0.049		
2nd-generation cephalosporin	0.799			0.289		
3rd-generation cephalosporin	0.235			0.870		
4th-generation cephalosporin	0.825			0.391		
Clindamycin	0.024	0.016	0.07 (0.007–0.60)	-		
Metronidazole	0.835			1.000		
Polymyxin B	-			0.790		
Meropenem	0.093			0.875		
Macrolides	0.580			0.078	[0.998]	[3.89 (0–Inf)]
**Monoinfection**	0.262			0.629		
**Carbapenemase genes**						
OXA-23	0.925			-		
OXA-51	0.352			-		
OXA-58	0.925			-		
OXA-48	-			0.873		
NDM	-			0.867		
No carbapenemase	-			0.930		
**Empiric antibiotics** **(Total infected patients: CRAB = 54; CRKP = 16)**						
Appropriate treatment	0.380			0.133		
**Definitive Treatment** **(Total infected patients: CRAB = 54; CRKP = 16)**						
Appropriate treatment	0.535			0.131		
Polymyxin B monotherapy	0.180			0.396		
Polymyxin B and Unasyn therapy	0.382			-		
Polymyxin B and Meropenem therapy	0.744			0.642		
Polymyxin B and other therapy	0.833			-		

Remarks: values in brankets [] indicate results before excluding high-probability variables. CKD: chronic kidney disease; DM: diabetes mellitus; CVD: cardiovascular disease; HPT: hypertension; Inf: infinity.

**Table 3:** Antimicrobial susceptibility profile for CRAB and CRKP strains.

Antibiotics	CRAB (n = 84)	CRKP (n = 62)
Amoxicillin-clavulanate (AMC)	-	62 (100.00%)
Ampicillin (AMP)	-	62 (100.00%)
Amikacin (AN)	70 (83.33%)	9 (14.52%)
Ceftazidime (CAZ)	82 (97.62%)	61 (98.39%)
Ciprofloxacin (CIP)	75 (89.29%)	51 (82.26%)
Ceftriaxone (CRO)	82 (97.62%)	61 (98.39%)
Cefotaxime (CTX)	83 (98.81%)	61 (98.39%)
Cefuroxime (CXM)	-	61 (98.39%)
Cefuroxime Axetil (CXMA)	-	26 (41.94%)
Ertapenem (ETP)	66 (78.57%)	59 (95.16%)
Cefepime (FEP)	84 (100.00%)	28 (45.16%)
Cefoxitin (FOX)	-	26 (41.94%)
Gentamicin (GM)	71 (84.52%)	13 (20.97%)
Imipenem (IPM)	84 (100.00%)	47 (75.81%)
Meropenem (MEM)	84 (100.00%)	50 (80.65%)
Ampicillin-sulbactam (SAM)	84 (100.00%)	62 (100.00%)
Trimethoprim-sulfamethoxazole (SXT)	54 (64.29%)	46 (74.19%)
Piperacillin-tazobactam (TZP)	84 (100.00%)	62 (100.00%)

## Data Availability

Data are contained within the article.
